# The comparative short-term efficacy and safety of drug-coated balloon vs. drug-eluting stent for treating small-vessel coronary artery lesions in diabetic patients

**DOI:** 10.3389/fpubh.2022.1036766

**Published:** 2022-10-18

**Authors:** Kui Li, Kaijun Cui, Xuechuan Dan, Jian Feng, Xiaobo Pu

**Affiliations:** ^1^Department of Cardiology, West China Hospital of Sichuan University, Chengdu, China; ^2^Department of Cardiology, The Second People's Hospital of Yibin, Yibin, China; ^3^Department of Cardiology, Affiliated Hospital of Southwest Medical University, Luzhou, China

**Keywords:** small-vessel coronary artery, diabetes mellitus, drug-coated balloon, drug-eluting stent, meta-analysis

## Abstract

**Purpose:**

This meta-analysis aimed to explore the comparative short-term efficacy and safety of drug-coated balloon (DCB) vs. drug-eluting stent (DES) for treating small-vessel coronary artery lesions in diabetic patients.

**Methods:**

We searched PubMed, EMBASE, the Cochrane Library, and China National Knowledgement Infrastructure (CNKI) for retrieving relevant studies regarding the comparison of DCB with DES in treating small-vessel coronary artery lesions in diabetic patients until May 31, 2022. Two independent authors screened study, extracted data, and assessed methodological quality. Then, the meta-analysis was conducted using RevMan software, version 5.4.

**Results:**

We included 6 studies with 847 patients in this meta-analysis. Pooled results showed that DCB was associated with fewer major adverse cardiac events (MACE) [RR, 0.60; 95% confidence interval (CI), 0.39–0.93; *p* = 0.02], myocardial infarction (MI) (RR, 0.42; 95% CI, 0.19–0.94; *p* = 0.03), target lesion revascularization (TLR) (RR, 0.24; 95% CI, 0.08–0.69; *p* < 0.001), target vessel revascularization (TVR) (RR, 0.33; 95% CI, 0.18–0.63; *p* < 0.001), binary restenosis (RR, 0.27; 95% CI, 0.11–0.68; *p* = 0.005), and late lumen loss (LLL) [mean difference (MD), −0.31; 95% CI, −0.36 to −0.27; *p* < 0.001], but was comparable technique success rate, death, minimal lumen diameter (MLD), and net lumen gain (NLG) to DES. There was no difference in long-term outcomes between these two techniques.

**Conclusions:**

This meta-analysis shows that DCB is better than DES in the short-term therapeutic efficacy and safety of small-vessel coronary artery lesions in diabetic patients. However, more studies are required to validate our findings and investigate the long-term effects and safety of DCB.

## Introduction

Patients who received percutaneous coronary interventions (PCI) usually present small-vessel coronary artery lesions, reporting an incidence of about 40% (1). Although significant advancements in therapeutic techniques, it remains challenging to treat small vessel coronary artery lesions resulting from a higher risk of technical failure, restenosis, and need for repeated revascularization (2, 3). Compared with non-diabetic patients, patients with diabetes mellitus suffered from worse clinical outcomes (e.g., binary restenosis and myocardial infarction) after PCI (4–7) owing to more challenging coronary anatomies (8–10), such as diffuse atherosclerotic plaques and higher frequency of thin-cap fibroatheroma and fibrocalcific atheroma (11).

The drug-eluting stent (DES) remains the cornerstone treatment for small-vessel coronary artery lesions (12) by reducing angiographic and clinical restenosis (13, 14). However, the presence of diabetes mellites significantly increases the risk of adverse outcomes as a significant predictor (15–18) because more stents of longer lengths and smaller diameters were usually required for PCI in diabetic patients (19). Therefore, the need to develop newer devices as alternatives to DES has been emphasized. As a result, drug-coated balloons (DCB) have attracted physicians' attention as a promising therapeutic modality for *de novo* lesions and small-vessel coronary artery lesions because they can deliver the antiproliferative drugs directly into the artery wall without the need for implanting metallic stents in the artery vessels (20).

Currently, several clinical trials and meta-analyses have evaluated the therapeutic role of DCB in treating small-vessel coronary artery lesions, indicating that the therapeutic efficacy and safety of DCB were not inferior to DES (21–25). However, only the meta-analysis by Razzack et al. (23) attempted to evaluate the therapeutic value of DCB in diabetic patients by introducing a subgroup analysis. Notably, this subgroup analysis involved only 3 eligible studies, which provided limited data to investigate the difference between DCB and DES in the treatment of small vessel coronary artery disease in diabetic patients. Meanwhile, most studies were underpowered to evaluate the differences between the DCB and DES in therapeutic efficacy and safety due to limited sample size (26–29). Therefore, we conducted this meta-analysis to investigate the comparative short-term therapeutic efficacy and safety of DCB vs. DES in diabetic patients with small-vessel coronary artery lesions.

## Methods

We first designed this meta-analysis's methodological framework, referring to the Cochrane handbook for systematic reviewers (30). Finally, we reported the meta-analysis's results according to the Preferred Reporting Items for Systematic Reviews and Meta-Analyses (PRISMA) checklist (31). The present study did not require institutional review or patient's informed consent because it was a meta-analysis of published data. However, we must point out that the formal protocol of this meta-analysis was not registered on a public platform.

### Search strategy

We systematically searched relevant studies on PubMed, EMBASE, and the Cochrane Library from their establishment date until May 31, 2022. We used the following major terms and their analogs to develop the basic search strategy, including “Coronary,” “diabetes,” “drug-eluting stent,” and “drug-eluting balloon.” We modified the basic search strategy to meet the requirements of each database. The detailed search strategy of each target database is summarized in Supplementary Table S1. In addition, we also checked the reference lists of studies included in this meta-analysis to identify those missing from the electronic literature search.

### Selection criteria

Two independent authors conducted the study selection by screening the titles, abstracts, and full texts of all retrieved studies according to the selection criteria were as follows: (1) Diabetic patients with small-vessel coronary artery lesions were treated with DCB or DES; (2) Studies reported at least one of the major adverse cardiac events (MACE) outcome, technique success rate, binary restenosis, minimal lumen diameter (MLD), late lumen loss (LLL), and net lumen gain (NLG); and (3) studies were published in English and Chinese, with full texts. We excluded ineligible studies following the exclusion criteria: (1) ineligible study designs, including case reports, experimental studies, reviews, and letters; (2) repeated publications of the same study; (3) essential data were not available after contacting the leading authors.

### Definition of outcomes

We defined the MACE as the primary endpoint, which was a composite outcome involving myocardial infarction (MI), target lesion revascularization (TLR), target vessel revascularization (TVR), and death (32). In addition, we defined technique success rate, binary restenosis, MLD, LLL, and NLG as the secondary endpoints. TLR and TVR are treated as two separate outcomes in this meta-analysis; however, TLR is part of TVR. Specifically, TLR was defined as repeated PCI treatment within the target lesion stent or edge 5 mm, but TVR was defined as PCI in the target lesion coronary vessel outside the stent (33). All outcomes were reported within 12 months after treatment, which were used to reveal short-term therapeutic efficacy and safety.

### Data extraction

Two independent authors conducted data extraction using the pre-designed standard information extraction sheet. The following data were extracted from all studies included in this meta-analysis, including the first author's name, publication year, country, study duration, study design, details of comparisons, sample size with the proportion of male patients, patients' mean age, basic reference vessel diameter (RVD), lesion length, diameter stenosis, the number of patients identified with American Heart Association (AHA) type B2/C lesion, and the information on the risk of bias. We calculated the transformed standard deviation (SD) based on the recognized formula (34) when the eligible study reported results as the interquartile range (IQR).

### Methodological quality assessment

Two authors assessed the methodological quality of all retrieved studies using the Cochrane risk of bias assessment tool version 2.0 (RoB 2.0) (35). Specifically, the RoB 2.0 quantified the overall methodological quality of a study from five areas, including “randomization process,” “deviations from the intended interventions,” “missing outcome data,” “measurement of the outcome,” and “selection of the reported result.” Using the RoB 2.0 tool, the overall methodological quality of one study was labeled with “low,” “high,” or “some concerns.” The results of the risk of bias assessment were graphically presented using the “robvis” command (36).

### Data analysis

For dichotomous variables, including the MACE outcome including MI, TLR, TVR, and death, technique success rate, and binary restenosis, relative risk (RR) with a 95% confidence interval (CI) was used to express the pooled estimate; however, for continuous variables, including MLD, LLL, and NLG, mean difference (MD) with a 95% CI was used to express the pooled estimate (37). We first tested the level of the statistical heterogeneity across studies using the Cochrane Q test (38) and I^2^ statistic (39). Significant heterogeneity was considered if *p* < 0.1 and I^2^ ≥ 50% (40) and random-effects model was selected for meta-analysis. On the contrary, the fixed-effects model was selected for meta-analysis when *p* > 0.1 and I^2^ < 50% (40). The publication bias examination was not conducted because only six eligible studies were included in this meta-analysis, which did not meet the criteria for constructing a funnel plot (41). Meta-analysis was conducted using RevMan software, version 5.4 (The Cochrane Collaboration, Copenhagen, Denmark) (42, 43).

## Results

### Study search

**Figure 1:**
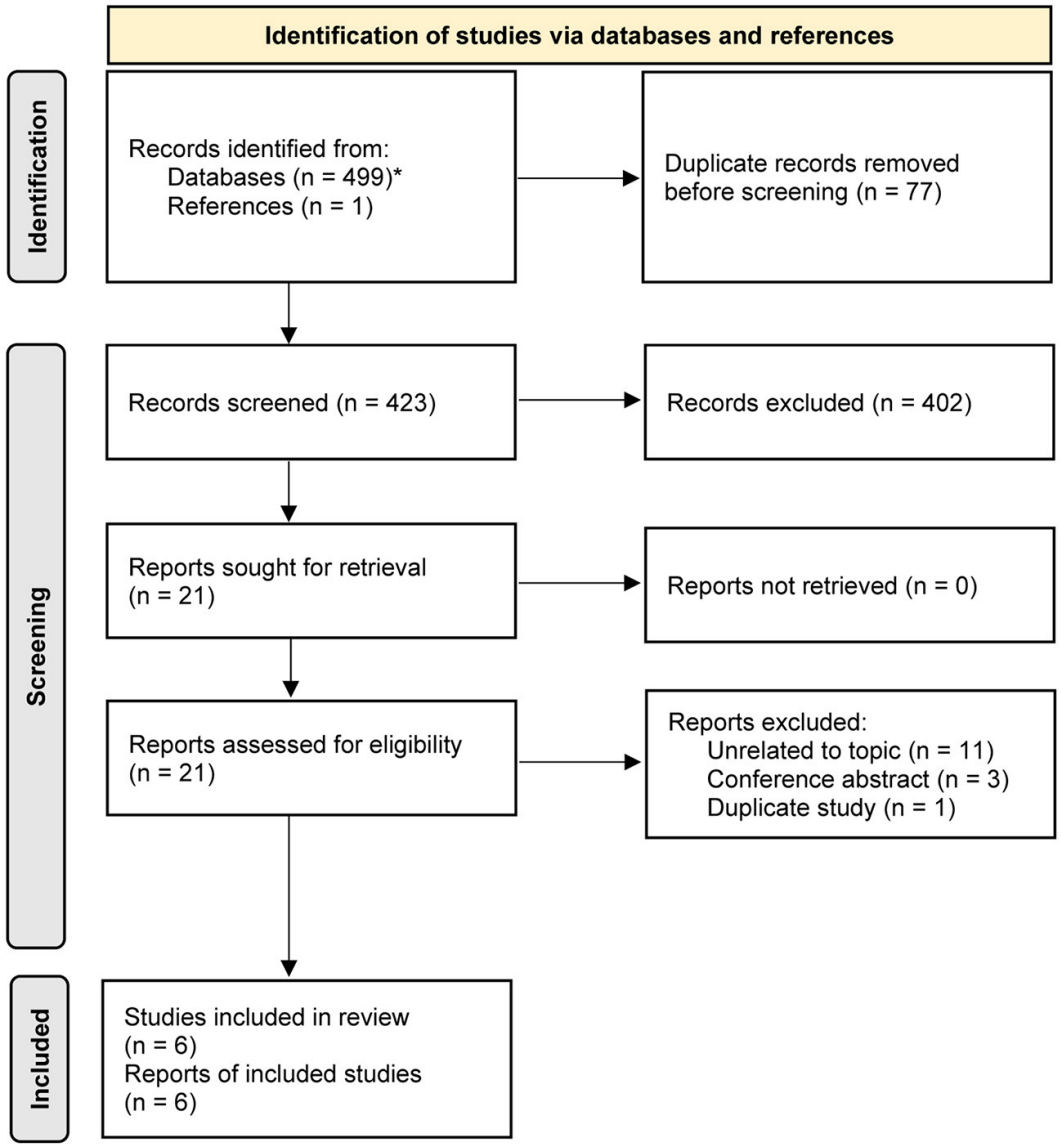
PRISMA flow chart of selecting study.

We retrieved 499 studies from the electronic literature search, and one additional study was identified from the reference list. Using the EndNote software, 77 duplicate studies were removed. After screening the titles and abstracts of 423 retaining studies, we excluded 402 ineligible studies. We accessed and screened the full texts of 21 studies, and 15 studies were excluded due to three reasons, including unrelated to the topic (*n* = 11), conference abstract without sufficient data (*n* = 3), and duplicate publication (*n* = 1). Finally, as shown in Figure 1, we included 6 studies (26–29, 44, 45) in this study for meta-analysis.

### Characteristics of studies

All studies were randomized controlled trials (RCTs) and were published between 2017 and 2021. Three studies were conducted in China (27–29), two studies in Italy (26, 44), and one study in Germany (45). Three studies (26, 44, 45) were conducted in multiple centers; however, other three studies (27–29) were conducted in a single center. The sample size of individual study ranged from 70 to 252, with an accumulated number of 847. Among the 6 included studies, five studies (26, 27, 29, 44, 45) reported MACE outcome, all studies (26–29, 44, 45) reported technique success, two studies (26, 27) reported binary restenosis, four studies (26–29) reported MLD and LLL, and three studies (26, 27, 29) reported NLG. We can access the remaining basic information of all studies in Table 1.

**Table 1:** Baseline information of 6 studies included in this meta-analysis.

**References**	**Country**	**Design**	**Criteria of small vessel**	**Group**	**Sample (male%)**	**Mean age, years**	**RVD, mm**	**Lesion length, mm**	**Diameter stenosis, %**	**AHA type B2/C lesion, n**	**Follow-up duration**
Giannini et al. (26)	Italy	Multicenter	RVD < 2.8 mm by visual estimation	PDEB	39 (82.05)	66.0	2.4 ± 0.4	15.3 ± 7.0	83 ± 10	25	12 months
				PES	35 (80.00)	70.0	2.5 ± 0.2	13.9 ± 5.0	84 ± 8	14	
Cortese et al. (44)	Italy	Multicenter	A vessel with a diameter between 2.00 and 2.75 mm with a target lesion ≥ 70%	PDEB	118 (70.34)	64.0	2.2 ± 0.4	13.5 ± 7.3	75 ± 17	n.r.	12 months
				EES	114 (76.32)	66.0	2.2 ± 0.4	14.0 ± 6.9	76 ± 15	n.r.	
Wöhrle et al. (45)	Germany	Multicenter	a small coronary vessel with a diameter between 2 and 3 mm	PDEB	122 (n.r.)	69.9	n.r.	n.r.	n.r.	n.r.	36 months
				EES	130 (n.r.)		n.r.	n.r.	n.r.	n.r.	
Zheng et al. (28)	China	Single-center	A vessel with a diameter between 2.25 and 2.80 mm with a target lesion ≥ 70%	PDEB	58 (75.86)	70.5	n.r.	n.r.	n.r.	n.r.	12 months
				PES	62 (77.42)	71.0	n.r.	n.r.	n.r.	n.r.	
Tang et al. (27)	China	Single-center	A vessel with a diameter < 2.80 mm with a target lesion ≥ 70%	PDEB	36 (69.44)	65.8	n.r.	n.r.	n.r.	n.r.	9 months
				PES	35 (65.71)	67.1	n.r.	n.r.	n.r.	n.r.	
Zhou et al. (29)	China	Single-center	A vessel with a diameter < 2.80 mm with a target lesion ≥ 70%	PDEB	50 (74.00)	61.9	n.r.	16.4 ± 5.5	n.r.	n.r.	12 months
				PES	48 (75.00)	62.4	n.r.	15.4 ± 5.8	n.r.	n.r.	

PDEB, paclitaxel drug-eluting balloon; PES, paclitaxel-eluting stent; EES, everolimus-eluting stent; RVD, reference vessel diameter; AHA, American Heart Association; DS, diameter stenosis; n.r., not reported.

### Risk of bias assessment

**Figure 2:**
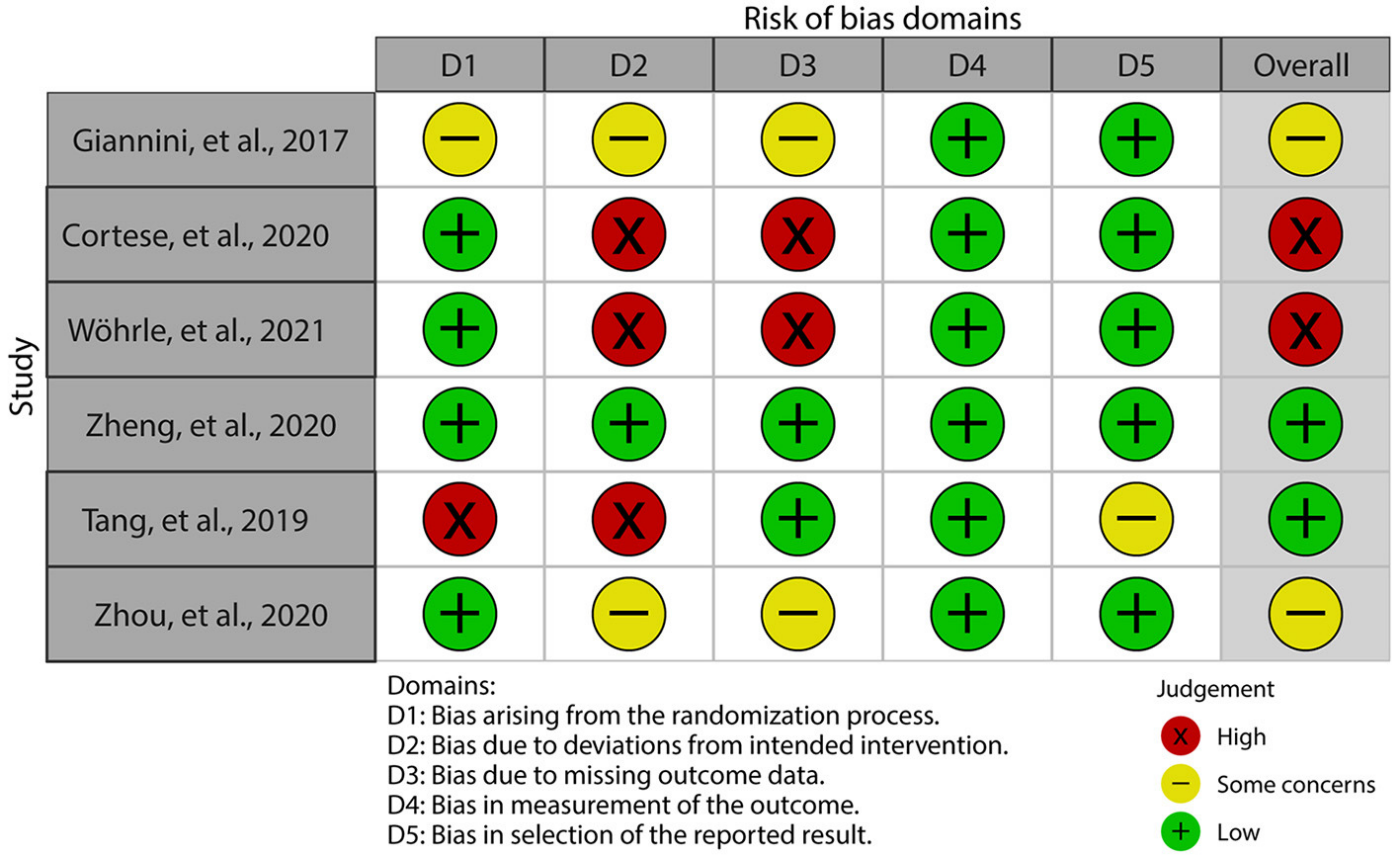
Risk of bias summary based on RoB 2.0. RoB, risk of bias.

One study was high risk in the randomization process, three studies were high risk in the deviations from intended interventions, two studies were high risk in the missing outcome data, and all studies were low or some concerns in the remaining two domains. Finally, the overall methodological quality was rated to be low to moderate. The results of the risk of bias assessment are depicted in Figure 2.

### Meta-analysis of MACE outcome

**Figure 3:**
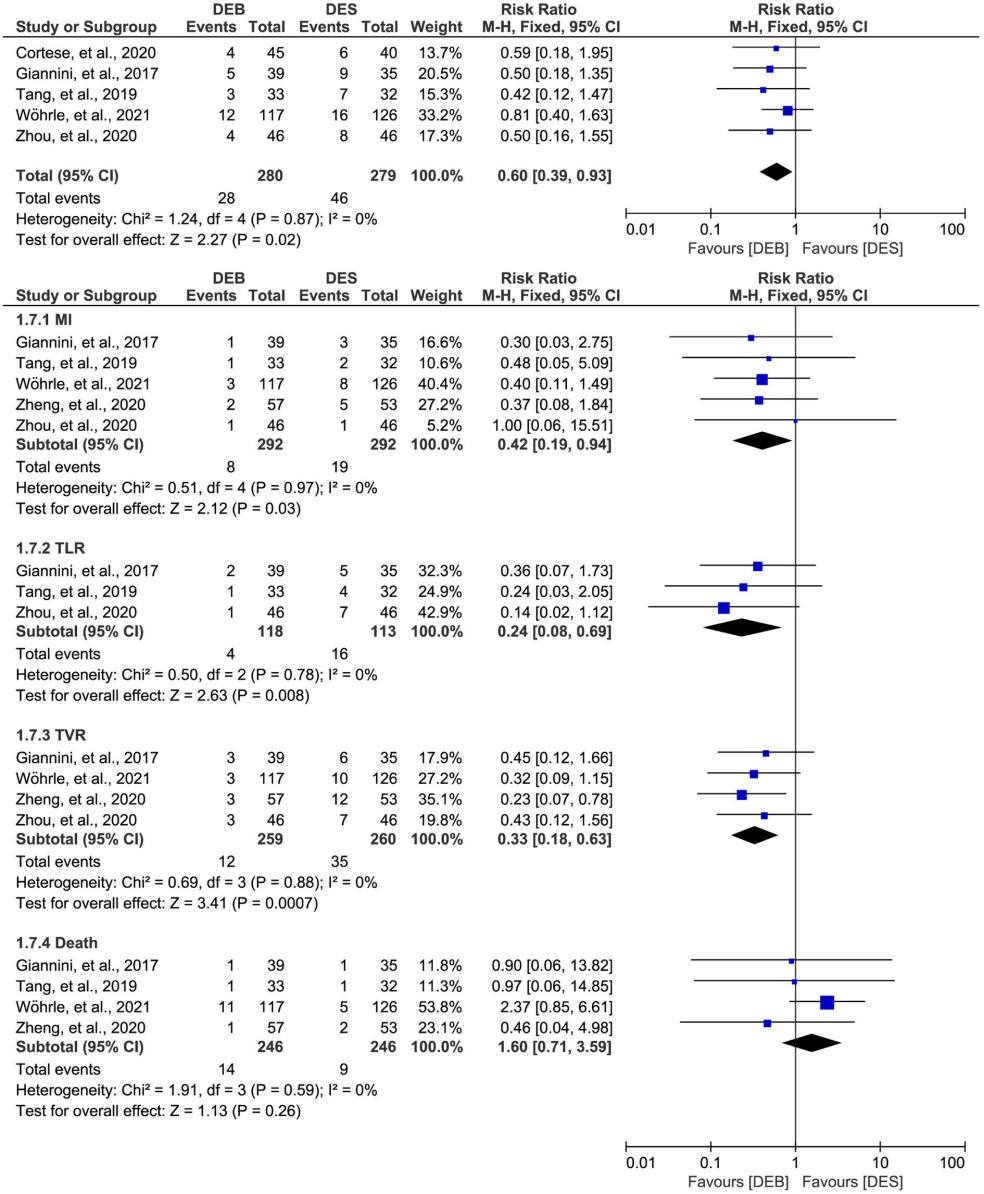
Meta-analysis of the MACE outcome. The black diamond represents the pooled result. If the black diamonds are completely to the left of the null line (“1”), it means that DEB is better than DES in terms of MACE results, MI, TLR, TVR, and death; if the black diamonds are completely to the right of the null line (“1”), it means that DEB is inferior to DES in terms of all outcomes; and if the black diamonds crossed through the null line (“1”), it means that DEB is comparable to DES in terms of all outcomes. MACE, major adverse cardiac events; MI, myocardial infarction; TLR, target lesion revascularization; TVR, target vessel revascularization; DCB, drug-eluting balloon; DES, drug-eluting stent; M-H, Mantel-Haenszel.

Among the 6 studies included in this meta-analysis, five studies (26, 27, 29, 44, 45) reported the data on the MACE outcome. There was no significant statistical heterogeneity across studies (*p* = 0.87, I^2^ = 0%), so we selected a fixed-effects model for meta-analysis. The meta-analysis suggested that DCB was associated with a decreased risk of MACE outcome compared to DES (RR, 0.60; 95% CI, 0.39–0.93; *p* = 0.02; Figure 3).

Furthermore, we conducted a subgroup analysis to investigate the difference between DCB and DES in a single MACE outcome. There was no significant statistical heterogeneity across studies, so we selected a fixed-effects model for meta-analysis. The results of subgroup analysis suggested significant difference between the two techniques in MI (RR, 0.42; 95% CI, 0.19–0.94; *p* = 0.03), TLR (RR, 0.24; 95% CI, 0.08–0.69; *p* = 0.008), and TVR (RR, 0.33; 95% CI, 0.18–0.63; *p* = 0.007), but not in death (RR, 1.60; 95% CI, 0.71–3.59; *p* = 0.26).

In addition, one study (45) also reported the MACE outcome at the 3-years follow-up. However, as shown in Supplementary Figure S1, there was no statistical difference between the techniques regarding the MACE outcome (RR, 0.87; 95% CI, 0.52–1.45; *p* = 0.59) and the single MACE outcome, including MI (RR, 0.65; 95% CI, 0.27–1.60; *p* = 0.35), TVR (RR, 0.56; 95% CI, 0.26–1.19; *p* = 0.13), and death (RR, 1.27; 95% CI, 0.67–2.42; *p* = 0.47).

### Meta-analysis of technique success and binary restenosis

**Figure 4:**
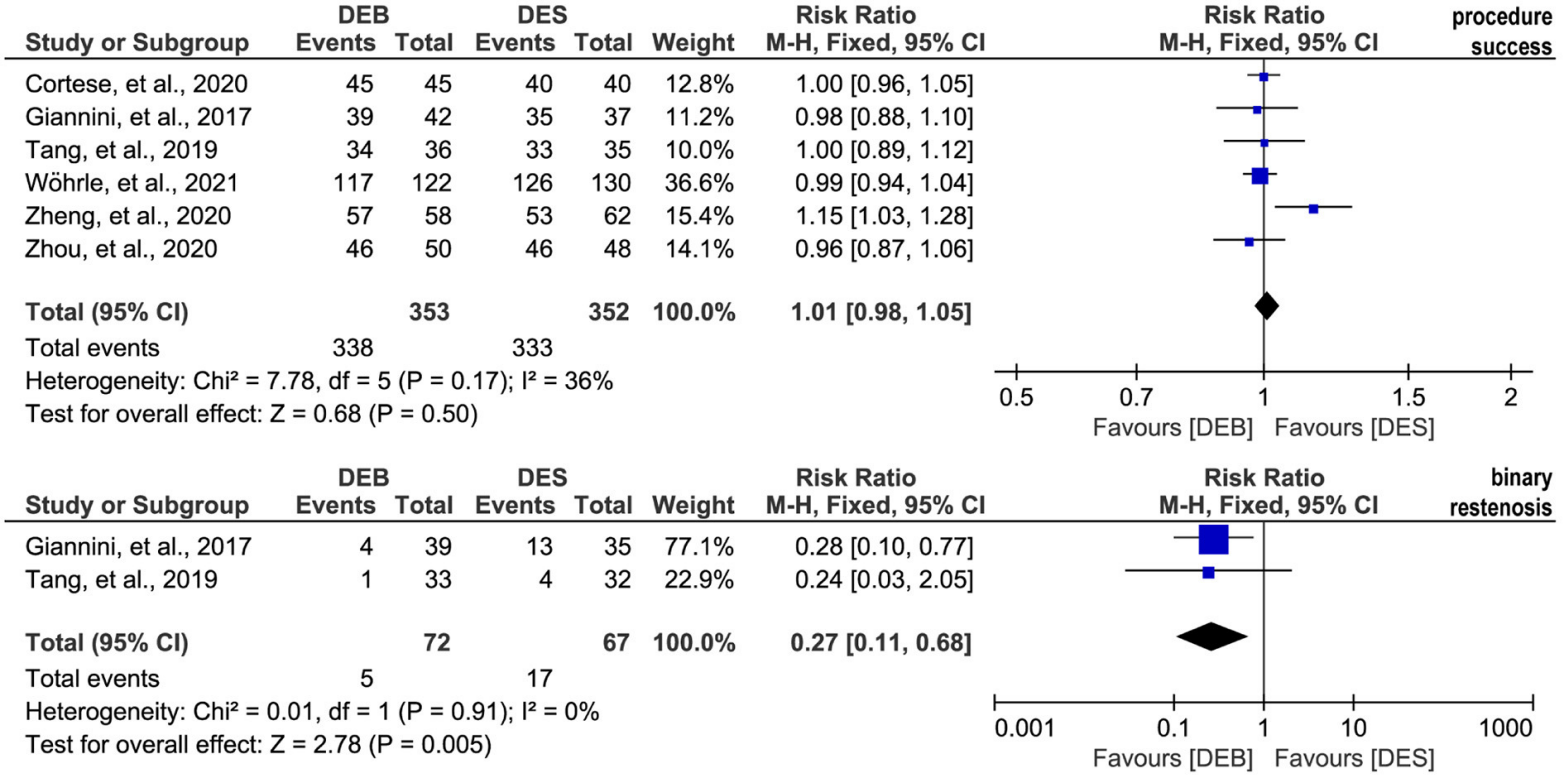
Meta-analysis of the procedure success rate and binary restenosis. The black diamond represents the pooled result. If the black diamonds are completely to the left of the null line (“1”), it means that DEB is better than DES in terms of the procedure success and binary restenosis; if the black diamonds are completely to the right of the null line (“1”), it means that DEB is inferior to DES in terms of all outcomes; and if the black diamonds crossed through the null line (“1”), it means that DEB is comparable to DES in terms of all outcomes. DCB, drug-eluting balloon; DES, drug-eluting stent; M-H, Mantel-Haenszel.

All included studies (26–29, 44, 45) reported the data on the technique success rate, and there was no significant statistical heterogeneity across studies (*p* = 0.17, I^2^ = 36%). Therefore, we selected a fixed-effects model for meta-analysis, and the pooled result suggested a comparable technique success rate between the two techniques (RR, 1.01; 95% CI, 0.98–1.05; *p* = 0.50; Figure 4). Moreover, two studies (26, 27) reported the data on the binary restenosis. There was no significant statistical heterogeneity across studies (*p* = 0.91, I^2^ = 0%), so we selected a fixed-effects model for meta-analysis. The pooled result suggested that DCB was associated with a lower binary restenosis rate than DES (RR, 0.27; 95% CI, 0.11–0.68; *p* = 0.005; Figure 4).

### Meta-analysis of MLD, LLL, and NLG

**Figure 5:**
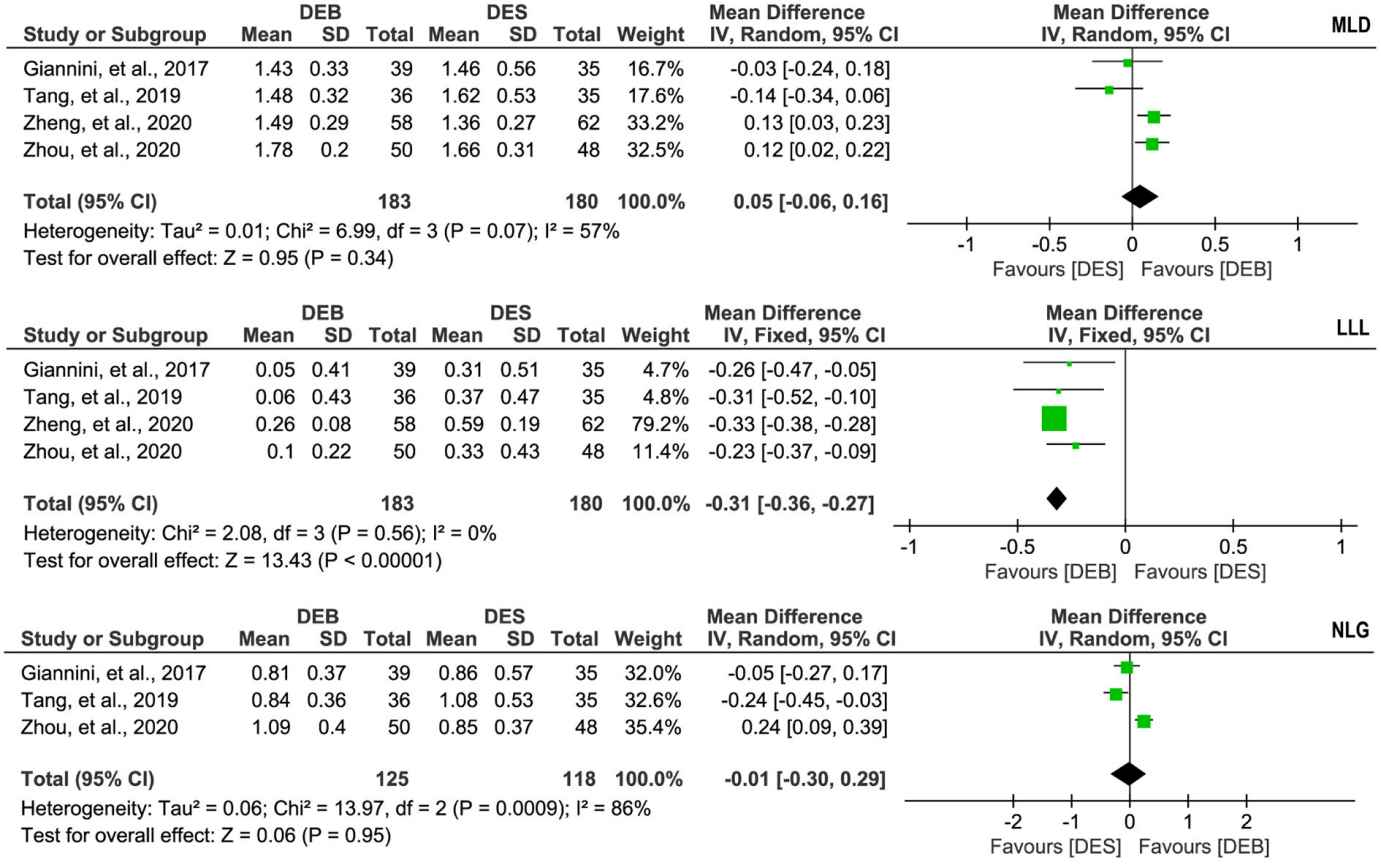
Meta-analysis of MLD, LLL, and NLG. The black diamond represents the pooled result. If the black diamonds are completely to the left of the null line (“0”), it means that DEB is better than DES in terms of MLD, LLL, and NLG; if the black diamonds are completely to the right of the null line (“0”), it means that DEB is inferior to DES in terms of all outcomes; and if the black diamonds crossed through the null line (“0”), it means that DEB is comparable to DES in terms of all outcomes. MLD, minimal lumen diameter; LLL, late lumen loss; NLG, net lumen gain; DCB, drug-eluting balloon; DES, drug-eluting stent; IV, inverse variance; SD, standard deviation.

Four studies (26–29) reported the data on the MLD, and there was significant statistical heterogeneity across studies (*p* = 0.07, I^2^ = 57%). Therefore, we selected a random-effects model for meta-analysis, and the pooled result suggested no statistical difference between the two techniques in the MLD (MD, 0.05; 95% CI, −0.06 to 0.16; *p* = 0.34, Figure 5). The same four studies (26–29) also reported the data on the LLL, and there was no significant statistical heterogeneity across studies (*p* = 0.56, I^2^ = 0%). The result of the meta-analysis based on a fixed-effects model suggested that patients receiving DCB had fewer LLL than patients treated by DES (MD, −0.31; 95% CI, −0.36 to −0.27; *p* < 0.001; Figure 5). In addition, three studies (26, 27, 29) reported the data on the NLG. There was significant statistical heterogeneity across studies (*p* < 0.1, I^2^ = 86%), so we selected a random-effects model for meta-analysis. The pooled result suggested no statistical difference between the two techniques regarding the NLG (MD, −0.01; 95% CI, −0.30 to 0.29; *p* = 0.95; Figure 5).

## Discussion

In the present meta-analysis, we systematically retrieved all relevant studies comparing DCB with DES in small-vessel coronary artery lesions among patients with diabetes mellitus. In the final data analysis, we included 6 low to moderate quality studies, accumulating a total of 847 patients. The pooled results showed that DCB was comparable to DES regarding technique success rate, MLD, and NLG; however, DCB had a lower risk in MACE outcome and binary restenosis. Subgroup analysis further indicated that DCB had a lower incidence in MI, TLR, and TVR than DES but comparable death to DES. The result from only one study suggested comparable MACE outcomes between the techniques at 3-years follow-up.

Interventional treatment of small-vessel coronary artery lesions is still challenging due to an increased risk of technical failure, restenosis, and the need for repeated revascularization (24), which is especially prominent in diabetic patients (11). DES remains the normative therapeutic strategy for PCI (46); however, implantation of DES will cause arterial wall injury to initiate vascular-proliferative cascade with smooth muscle cell proliferation and migration, resulting in neointimal hyperplasia (47). Compared to DES, DCB can deliver the antiproliferative drug into the vessel wall without the need for the implantation of metal struts, therefore directly inhibiting endothelial proliferation and adverse remodeling (20). From the theoretical perspective, the implantation of DCB will be superior to DES for treating small-vessel coronary artery lesions, which also interprets why the present meta-analysis found that DCB was associated with fewer binary restenosis, LLL, and single MACE outcome.

Currently, several studies (21–25) have investigated the comparative efficacy and safety of DCB vs. DES for treating *de novo* lesions in small-vessel coronary disease using the meta-analytic technique. However, the meta-analyses by Li et al. (21) did not isolate diabetic patients from general populations, although authors found that DCB was non-inferior to DES, delivering a good outcome in non-fatal MI, and can be recommended as an optimal treatment strategy in patients with *de novo* small-vessel coronary artery diseases. In addition, the meta-analysis by Elgendy et al. (22) assessed the differences in reducing TLR between DCB and DES in novo small-vessel coronary artery by introducing subgroup analysis; however, this meta-analysis did not also isolate diabetic patients form general populations. Another meta-analysis by Razzack et al. (23) included eight studies first to investigate the difference in therapeutic efficacy and safety between DCB and DES in treating *de novo* lesions in small-vessel coronary disease. Then, the authors evaluated the therapeutic value of DCB in diabetic patients by introducing a subgroup analysis involving 3 studies, indicating no statistical difference between DCB and DES regarding the MACE outcome [odds ratio (OR), 1.34; 95% CI, 0.73–2.46; *p* = 0.34], inconsistent with our finding.

In the present meta-analysis, we specifically evaluated the therapeutic efficacy and safety of DCB vs. DES for treating small-vessel coronary artery lesions in diabetic patients. The results of our meta-analysis provided more specific evidence-based information for practitioners dedicated to treating small coronary vessel lesions in diabetic patients compared with that meta-analyses reported by Li et al. (21) and Elgendy et al. (22). In addition, 6 eligible studies were included in our meta-analysis. Therefore, the statistical power of this meta-analysis was significantly higher than the meta-analysis by Razzack et al. (23), generating more reliable results. As a result, we can have the confidence to convince that DCB is associated with fewer MACE outcomes than DES in treating small-vessel coronary artery lesions in diabetic patients. More importantly, the present meta-analysis not only included the MACE outcome, but also considered other outcomes, including technique success rate, binary restenosis, MLD, LLL, and NLG, which benefited us to evaluate the therapeutic efficacy and safety of DCB more comprehensively for small-vessel coronary artery lesions in diabetic patients.

Although this meta-analysis included RCTs to enhance the reliability of the pooled results, we cannot ignore that it faced some limitations. First, although 6 eligible studies were included in the final analysis, not all studies reported all outcomes. Therefore, studies included for individual outcome remains limit, which may adversely impact the robustness of the pooled results. Second, the results of the risk of bias assessment suggested that the overall methodological quality of 6 included studies was low to moderate. Therefore, we cannot eliminate the negative impact of low methodological quality on the robustness of the pooled results. Third, we detected significant statistical heterogeneity for meta-analyses of some outcomes. However, we could not conduct a sensitivity analysis to test the robustness of the results due to limited studies. As a result, we should cautiously interpret the results with significant statistical heterogeneity. Fourth, only one study reported outcomes in the long-term follow-up; therefore, we could not adequately evaluate the differences in long-term therapeutic efficacy and safety between the two techniques. Fifth, we could not assess the potential differences in treatment effect among different type of DCB because limited data are available. Sixth, our finding should be interpreted with caution because the criteria of small vessel and the follow-up duration varied slightly between included studies (as shown in Table 1). Finally, the formal protocol of this meta-analysis was not registered publicly although we conducted it in strict accordance with the process of a meta-analysis.

In conclusion, the present meta-analysis suggested that DCB is better than DES in the short-term therapeutic efficacy and safety of small-vessel coronary artery lesions in diabetic patients because DCB can significantly decrease the LLL and reduce the risk of binary restenosis, and it is also associated with fewer risk of MI, TLR, and TVR. However, all findings of this meta-analysis are generated from studies with low to moderate quality. Meanwhile, only one study evaluates the long-term therapeutic efficacy and safety. Therefore, more multi-center, large-scale, and high-quality studies are needed to validate our findings and investigate the difference between the two techniques in the long-term outcomes.

## Data availability statement

The raw data supporting the conclusions of this article will be made available by the authors, without undue reservation.

## Author contributions

KL: conceptualization, formal analysis, and writing original draft preparation. KL and KC: methodology and validation. KC: resources and project administration. JF and XP: data curation. KL, JF, and XP: writing review and editing. All authors have read and agreed to the published version of the manuscript.

## Conflict of interest

The authors declare that the research was conducted in the absence of any commercial or financial relationships that could be construed as a potential conflict of interest.

## Publisher's note

All claims expressed in this article are solely those of the authors and do not necessarily represent those of their affiliated organizations, or those of the publisher, the editors and the reviewers. Any product that may be evaluated in this article, or claim that may be made by its manufacturer, is not guaranteed or endorsed by the publisher.

## Supplementary material

Supplementary Figure S1 Meta-analysis of the MACE outcome at 3-years follow-up. The black diamond represents the pooled result. If the black diamonds are completely to the left of the null line (“1”), it means that DEB is better than DES in terms of MACE outcome; if the black diamonds are completely to the right of the null line (“1”), it means that DEB is inferior to DES in terms of MACE outcome; and if the black diamonds crossed through the null line (“1”), it means that DEB is comparable to DES in terms of MACE outcome. MACE, major adverse cardiac events; MI, myocardial infarction; TVR, target vessel revascularization; DCB, drug-eluting balloon; DES, drug-eluting stent; M-H, Mantel-Haenszel.

Supplementary Table S1 Detailed search strategies of target databases.

## Abbreviations

PCI: percutaneous coronary interventions
DCB: drug-coated balloons
PRISMA: Preferred Reporting Items for Systematic Reviews and Meta-Analyses
MACE: major adverse cardiac events
MLD: minimal lumen diameter
LLL: late lumen loss
NLG: net lumen gain
MI: myocardial infarction
TLR: target lesion revascularization
TVR: target vessel revascularization.
