# Supplementary figures and images for: The comparative short-term efficacy and safety of drug-coated balloon vs. drug-eluting stent for treating small-vessel coronary artery lesions in diabetic patients
Source: Front Public Health. 2022 Oct 18;10:1036766. doi: 10.3389/fpubh.2022.1036766 (PMC9623093; doi:10.3389/fpubh.2022.1036766)

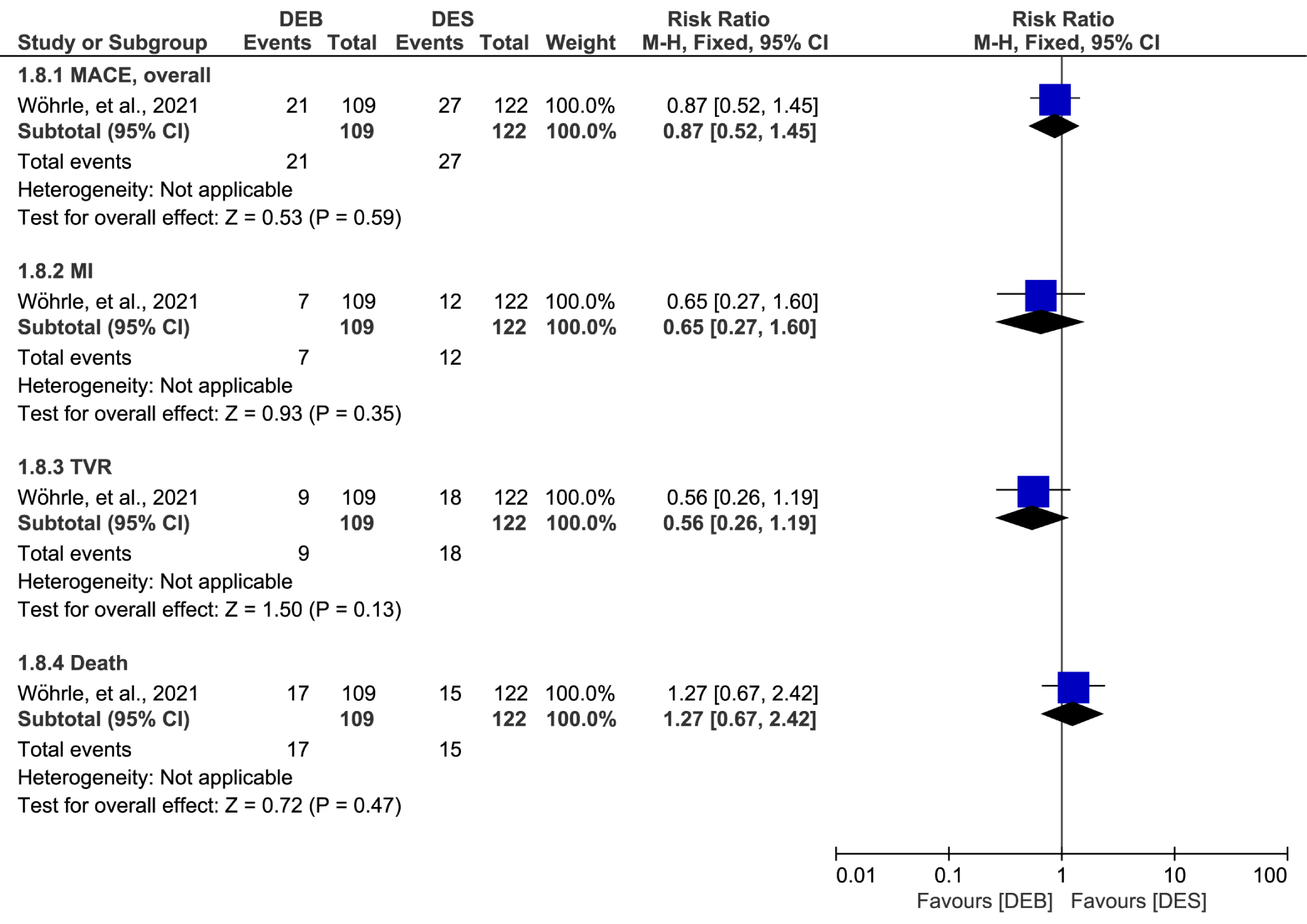

Supplement: Supplementary Figure S1 — Meta-analysis of the MACE outcome at 3-years follow-up. The black diamond represents the pooled result. If the black diamonds are completely to the left of the null line (“1”), it means that DEB is better than DES in terms of MACE outcome; if the black diamonds are completely to the right of the null line (“1”), it means that DEB is inferior to DES in terms of MACE outcome; and if the black diamonds crossed through the null line (“1”), it means that DEB is comparable to DES in terms of MACE outcome. MACE, major adverse cardiac events; MI, myocardial infarction; TVR, target vessel revascularization; DCB, drug-eluting balloon; DES, drug-eluting stent; M-H, Mantel-Haenszel. [file Image_1.JPEG]
