# Supplementary material for: The comparative short-term efficacy and safety of drug-coated balloon vs. drug-eluting stent for treating small-vessel coronary artery lesions in diabetic patients
Source: Front Public Health. 2022 Oct 18;10:1036766. doi: 10.3389/fpubh.2022.1036766 (PMC9623093; doi:10.3389/fpubh.2022.1036766)
Supplement: Supplementary Table S1 — Detailed search strategies of target databases. [file Table_1.DOCX]

**Table S1.** Detailed search strategy of target databases.

(I) Search strategy of PubMed

| No. | Search Details | Results |
| --- | --- | --- |
| 10 | (("Drug-Eluting Stents"[MeSH Terms] OR ("drug eluting stent"[Title/Abstract] OR "Drug-Eluting Stents"[Title/Abstract] OR "drug eluting stent"[Title/Abstract] OR "Drug-Eluting Stents"[Title/Abstract] OR "drug coated stents"[Title/Abstract] OR "drug coated stent"[Title/Abstract] OR "drug coated stents"[Title/Abstract] OR "drug coated stent"[Title/Abstract])) AND ("drug eluting balloon"[Title/Abstract] OR "drug eluting balloons"[Title/Abstract] OR "drug coated balloon"[Title/Abstract] OR "drug coated balloons"[Title/Abstract] OR "drug coated balloon"[Title/Abstract] OR "drug coated balloons"[Title/Abstract] OR "drug eluting balloon"[Title/Abstract] OR "drug eluting balloons"[Title/Abstract]) AND ("Diabetes Mellitus"[MeSH Terms] OR ("Diabetes"[Title/Abstract] OR "Diabetic"[Title/Abstract])) AND "Coronary"[Title/Abstract]) AND (humans[Filter]) | 46 |
| 9 | ("Drug-Eluting Stents"[MeSH Terms] OR ("drug eluting stent"[Title/Abstract] OR "Drug-Eluting Stents"[Title/Abstract] OR "drug eluting stent"[Title/Abstract] OR "Drug-Eluting Stents"[Title/Abstract] OR "drug coated stents"[Title/Abstract] OR "drug coated stent"[Title/Abstract] OR "drug coated stents"[Title/Abstract] OR "drug coated stent"[Title/Abstract])) AND ("drug eluting balloon"[Title/Abstract] OR "drug eluting balloons"[Title/Abstract] OR "drug coated balloon"[Title/Abstract] OR "drug coated balloons"[Title/Abstract] OR "drug coated balloon"[Title/Abstract] OR "drug coated balloons"[Title/Abstract] OR "drug eluting balloon"[Title/Abstract] OR "drug eluting balloons"[Title/Abstract]) AND ("Diabetes Mellitus"[MeSH Terms] OR ("Diabetes"[Title/Abstract] OR "Diabetic"[Title/Abstract])) AND "Coronary"[Title/Abstract] | 52 |
| 8 | "Coronary"[Title/Abstract] | 434,057 |
| 7 | "Diabetes Mellitus"[MeSH Terms] OR "Diabetes"[Title/Abstract] OR "Diabetic"[Title/Abstract] | 782,006 |
| 6 | "Diabetes"[Title/Abstract] OR "Diabetic"[Title/Abstract] | 716,426 |
| 5 | "Diabetes Mellitus"[MeSH Terms] | 480,435 |
| 4 | "drug eluting balloon"[Title/Abstract] OR "drug eluting balloons"[Title/Abstract] OR "drug coated balloon"[Title/Abstract] OR "drug coated balloons"[Title/Abstract] OR "drug coated balloon"[Title/Abstract] OR "drug coated balloons"[Title/Abstract] OR "drug eluting balloon"[Title/Abstract] OR "drug eluting balloons"[Title/Abstract] | 1,819 |
| 3 | "Drug-Eluting Stents"[MeSH Terms] OR "drug eluting stent"[Title/Abstract] OR "Drug-Eluting Stents"[Title/Abstract] OR "drug eluting stent"[Title/Abstract] OR "Drug-Eluting Stents"[Title/Abstract] OR "drug coated stents"[Title/Abstract] OR "drug coated stent"[Title/Abstract] OR "drug coated stents"[Title/Abstract] OR "drug coated stent"[Title/Abstract] | 17,997 |
| 2 | "drug eluting stent"[Title/Abstract] OR "drug eluting stents"[Title/Abstract] OR "drug eluting stent"[Title/Abstract] OR "drug eluting stents"[Title/Abstract] OR "drug coated stents"[Title/Abstract] OR "drug coated stent"[Title/Abstract] OR "drug coated stents"[Title/Abstract] OR "drug coated stent"[Title/Abstract] | 12,557 |
| 1 | "Drug-Eluting Stents"[MeSH Terms] | 13,180 |

(II) Search strategy of EMBASE

| No. | Query | Results |
| --- | --- | --- |
| #9 | #3 AND #4 AND #7 AND #8 | 197 |
| #8 | coronary:ti,ab,kw | 636964 |
| #7 | #5 OR #6 | 1306505 |
| #6 | 'diabetes mellitus'/exp | 1150327 |
| #5 | diabetes:ti,ab,kw | 947021 |
| #4 | 'drug eluting balloon':ti,ab,kw OR 'drug eluting balloons':ti,ab,kw OR 'drug-coated balloon':ti,ab,kw OR 'drug-coated balloons':ti,ab,kw OR 'drug coated balloon':ti,ab,kw OR 'drug coated balloons':ti,ab,kw OR 'drug-eluting balloon':ti,ab,kw OR 'drug-eluting balloons':ti,ab,kw | 3183 |
| #3 | #1 OR #2 | 39909 |
| #2 | 'drug eluting stent'/exp | 37995 |
| #1 | 'drug eluting stent':ti,ab,kw OR 'drug eluting stents':ti,ab,kw OR 'drug-eluting stent':ti,ab,kw OR 'drug-eluting stents':ti,ab,kw OR 'drug-coated stents':ti,ab,kw OR 'drug-coated stent':ti,ab,kw OR 'drug coated stents':ti,ab,kw OR 'drug coated stent':ti,ab,kw | 23263 |

(III) Search strategy of Cochrane library

| ID | Search | Hits |
| --- | --- | --- |
| #1 | (Drug Eluting Stent):ti,ab,kw OR (Drug Eluting Stents):ti,ab,kw OR (Drug-Eluting Stent):ti,ab,kw OR (Drug-Eluting Stents):ti,ab,kw OR (Drug-Coated Stents):ti,ab,kw | 4466 |
| #2 | (Drug-Coated Stent):ti,ab,kw OR (Drug Coated Stents):ti,ab,kw OR (Drug Coated Stent):ti,ab,kw | 834 |
| #3 | #1 or #2 | 4616 |
| #4 | MeSH descriptor: [Drug-Eluting Stents] explode all trees | 1575 |
| #5 | #3 or #4 | 4616 |
| #6 | (drug eluting balloon):ti,ab,kw OR (drug eluting balloon):ti,ab,kw OR (drug-coated balloon):ti,ab,kw OR (drug-coated balloons):ti,ab,kw OR (drug coated balloon):ti,ab,kw | 1608 |
| #7 | (drug coated balloon):ti,ab,kw OR (drug-eluting balloon):ti,ab,kw OR (drug-eluting balloons):ti,ab,kw | 1586 |
| #8 | #6 or #7 | 1634 |
| #9 | (Diabetes):ti,ab,kw OR ("diabetic"):ti,ab,kw | 103697 |
| #10 | MeSH descriptor: [Diabetes Mellitus] explode all trees | 35175 |
| #11 | #9 or #10 | 103987 |
| #12 | (coronary):ti,ab,kw | 61258 |
| #13 | #5 and #8 and #11 and #12 | 179 |
